# Printed educational materials directed at Ontario family physicians do not improve adherence to guideline recommendations for diabetes management: a pragmatic, factorial, cluster randomized controlled trial [ISRCTN72772651]

**DOI:** 10.1186/s12875-021-01592-9

**Published:** 2021-12-11

**Authors:** Alison H. Howie, Neil Klar, Danielle M. Nash, Jennifer N. Reid, Merrick Zwarenstein

**Affiliations:** 1 Department of Epidemiology and Biostatistics, Western Centre for Public Health and Family Medicine, 1465 Richmond St., London, ON N6G 2M1 Canada; 2 ICES, Toronto, ON Canada; 3 Department of Family Medicine, Western Centre for Public Health and Family Medicine, 1465 Richmond St, London, ON N6G 2M1 Canada

**Keywords:** Printed educational materials, Cluster randomized controlled trial, Pragmatic, Knowledge translation, Diabetes

## Abstract

**Background:**

Printed educational materials (PEMs) have long been used to inform clinicians on evidence-based practices. However, the evidence for their effects on patient care and outcomes is unclear. In Ontario, despite widely available clinical practice guidelines recommending antihypertensives and cholesterol-lowering agents for patients with diabetes, prescriptions remain low. We aimed to determine whether PEMs can influence physicians to intensify prescribing of these medications.

**Methods:**

A pragmatic, 2 × 2 factorial, cluster randomized controlled trial was designed to ascertain the effect of two PEM formats on physician prescribing: a postcard-sized message (“outsert”) or a longer narrative article (“insert”). Ontario family physician practices (clusters) were randomly allocated to receive the insert, outsert, both or neither. Physicians were eligible if they were in active practice and their patients were included if they were over 65 years with a diabetes diagnosis; both were unaware of the trial. Administrative databases at ICES (formerly the Institute for Clinical Evaluative Sciences) were used to link patients to their physician and to analyse prescribing patterns at baseline and 1 year following PEM mailout. The primary outcome was intensification defined as the addition of a new antihypertensive or cholesterol-lowering agent, or dose increase of a current drug, measured at the patient level. Analyses were by intention-to-treat and accounted for the clustering of patients to physicians.

**Results:**

We randomly assigned 4231 practices (39% of Ontario family physicians) with a total population of 185,526 patients (20% of patients with diabetes in Ontario primary care) to receive the insert, outsert, both, and neither; among these, 4118 practices were analysed (*n* = 1025, *n* = 1037, *n* = 1031, n = 1025, respectively). No significant treatment effect was found for the outsert (odds ratio (OR) 1.01, 95% confidence interval (CI) 0.98 to 1.04) or the insert (OR 0.99, 95% CI 0.96 to 1.02). Percent of intensification in the four arms was similar (approximately 46%). Adjustment for physician characteristics (e.g., age, sex, practice location) had no impact on these findings.

**Conclusions:**

PEMs have no effect on physician’s adherence to recommendations for the management of diabetes-related complications in Ontario. Further research should investigate the effect of other strategies to narrow this evidence-to-practice gap.

**Trial registration:**

ISRCTN72772651. Retrospectively registered 21 July 2005.

**Supplementary Information:**

The online version contains supplementary material available at 10.1186/s12875-021-01592-9.

## Background

Researchers have estimated that it takes 17 years for research findings to enter usual care [1]. Thus, clinicians may deliver outdated, unnecessary, and even harmful care many years after evidence emerges [2]. This disconnect between research and routine clinical practice is known as the evidence-to-practice gap, or the second translational gap, and prevents patients from receiving the best-known care [3].

Evidence-to-practice gaps are prominent in primary care [3]. For example, in Ontario, despite a universal health insurance system, medications proven to prevent diabetes-related complications are underprescribed [4]. Cardiovascular disease remains the leading cause of death and disability among individuals with diabetes [5], highlighting the need for knowledge translation (KT) strategies to improve the clinical management of its risk factors, such as hypertension. Antihypertensives and cholesterol-lowering agents have been shown to reduce morbidity in individuals with diabetes more effectively than glucose-lowering agents alone [6, 7]. Thus, a multi-faceted treatment approach, managing all risk factors, is vital. This body of evidence has been translated into numerous clinical practice guidelines [8–11]; nevertheless, in 2007, the prescribing of relevant medications in Ontario fell short of guideline recommendations by as much as 30% [4].

Many KT strategies have been proposed to help close such evidence-to-practice gaps. Printed educational materials (PEMs) are recommendations for clinical care delivered in print format to the recipient, and have long been favoured to address evidence-to-practice gaps due to their low costs and potential for wide reach [12]. Bero et al. were the first to review evaluations of this KT strategy, finding that PEMs have little to no effect on changing provider behaviour, and therefore recommended that more intensive interventions be used to alter practice [13]. However, in a 2004 systematic review, PEMs were found to result in modest absolute improvements in care, ranging from 3.6 to 17%, with a median of 8.1%; thus, the authors concluded that PEMs should not be discarded, especially as costs are low and, in many situations, resources for behaviour change interventions are scarce [14]. Clarifying this, a 2008 systematic review concluded that PEMs were ineffective when compared to other KT interventions, but when compared to no intervention, led to statistically significant improvements in care [15]. However, a 2015 systematic review again concluded that PEMs do not improve outcomes, neither at the physician level, nor at the patient level [16]. These authors suggested that the encouraging results identified in previous reviews may be due to the inclusion of specialist physicians, who might respond differently to PEMs than do primary care physicians [16]. Most recently, the evidence has again reversed, with a 2020 systematic review finding that PEMs have “modest, but potentially important, improvements in professional practice” [17]. The conclusions from this review were influenced by the findings from the first two published OPEM (Ontario Printed Educational Messages) trials [18, 19]. The third and last OPEM trial is reported here, providing more real-world randomized evidence on the effectiveness of PEMs.

### Aim

The aim of the OPEM programme was to test if PEMs improve care in a larger scale within usual primary care clinical practice. The underlying purpose was to help determine whether PEMs should be routinely used by local decision makers (i.e., the Ontario Ministry of Health and Long-Term Care) aiming to address evidence-to-practice gaps in primary care. We report here the third of three pragmatic [20], multicenter, factorial, cluster randomized controlled trials (cRCTs) amongst Ontario family physicians (FPs). A cluster design was chosen to minimize contamination between physicians working together, and between patients cared for by the same physician. Further, a factorial design allowed us to simultaneously test the independent effects of two different versions of a PEM: an insert and an outsert (described in detail in methods). In this randomized controlled trial (RCT), we aimed to establish whether PEMs urging intensification of antihypertensives and cholesterol-lowering medications had an impact on physician prescribing of medications for diabetes-related complications, measured at the individual patient level.

## Methods

### Study design and randomization

A pragmatic [20], multicenter, 2 × 2 factorial, cRCT design was chosen with physician practices as the unit of randomization and outcomes measured at the individual patient level. To prevent contamination, physicians who work in group or shared practices were identified by common address and were randomized to receive the same intervention [4]. Group practices allow for patients to be seen by more than one physician; therefore, patients who received a prescription written by an Ontario FP qualified for inclusion, even if the physician wasn’t their primary care provider [4].

Randomization was carried out at the cluster level by the study statistician using computer-generated random numbers, omitting stratification. Practices were allocated in a 1:1:1:1 ratio to receive a longer or shorter PEM, both, or neither. Allocation was concealed from physicians and patients who were both blinded as to the existence of the trial; physicians were obviously aware of the intervention they received but it is unlikely that patients were made aware of this by their physician. All patients who qualified for inclusion within the practices were automatically included in the study and their prescriptions analysed; no recruitment efforts were required.

The PEMs were mailed alongside the January edition of *informed* (see intervention section) to all Ontario FP practices on January 15, 2005. Pre- and post-intervention prescribing were measured from January 15, 2004 to January 14, 2005, and January 16, 2005 to January 15, 2006, respectively.

Additional details regarding the study design are available in the published protocol [4].

### Study setting

The study was carried out in Ontario, Canada. Ontario residents are eligible to access the majority of health care services at no cost as a result of a publicly funded health care system, the Ontario Health Insurance Program (OHIP) [21]. Among the list of qualifying services is visits to a family doctor [21]. Further, the majority of prescriptions are covered for Ontario residents aged 65 and older through the Ontario Drug Benefit (ODB) program [22]. This includes medications to treat diabetes [22].

### Data sources

All study data were obtained from administrative databases at ICES (formerly the Institute for Clinical Evaluative Sciences); no data collection or real time follow-up of participants was required. The following seven databases were linked to ascertain patient and physician characteristics, treatment regimens, and the effectiveness of the intervention: 1) Ontario Drug Benefit Claims (**ODB**, which collects prescription information for individuals 65 and older in Ontario) [23]; 2) Ontario Health Insurance Plan Claims Database (**OHIP**, which contains information on the inpatient and outpatient services that are publicly funded for Ontario residents); 3) Corporate Provider Database (**CPDB**, which contains demographic, specialty, eligibility, and practice location information for all physicians funded by the Ministry of Health); 4) ICES Physician Database (**IPDB**, which contains information on both demographic and professional characteristics of all Ontario physicians); 5) Registered Persons Database (**RPDB**, which houses information on basic patient demographics, received from the Ministry of Health, for all Ontarians with a health card number); 6) Drugs List (which contains the 8-digit drug identification number (**DIN**) for prescription drugs approved for use in Canada); 7) Ontario Diabetes Dataset (**ODD**, an ICES-derived database that contains information on incident and prevalent cases of diabetes in Ontario) [24]. These datasets were linked using unique, encoded identifiers and analyzed at ICES.

### Study population

#### Physicians

Eligible FPs were registered for reimbursement with OHIP between August 1, 2003 and July 31, 2004 (lookback period) [4]. At the time of the study, the vast majority of Ontario physicians billed through OHIP [25]. FPs who were not considered to be in “active” practice in 2004 were excluded. This included those who accumulated less than $50,000 in fee-for-service billings in 2004, those who prescribed medications to fewer than 100 patients aged 66 and above in 2004, and those who did not prescribe medication to an individual 66 or older in at least 10 months in 2004 [14]. The latter two exclusion criteria were used to ensure that the physicians had adequate experience with seniors. Furthermore, physicians who submitted a claim under a speciality other than FP during the lookback period were excluded. Physicians were excluded during the post-intervention period for the following reasons: submitting a claim under a speciality other than FP, no longer practicing, failed to be randomized, not matched to at least one patient with diabetes, missing information on personal identifiers (due to ICES constraints), and working in shared practices that were mistakenly sent multiple versions of the PEM.

#### Patients

Patients were considered for inclusion if they had a diabetes diagnosis (type 1 or type 2) on or before January 15, 2004. Individuals who were younger than 66 years as of January 15, 2005, had an invalid personal identifier at ICES, or were non-Ontario residents were excluded. Individuals who did not see an OPEM physician during the pre-intervention period, or received an equal number of billable services from more than one physician, were also excluded. Individuals who did not see an OPEM physician during the post-intervention period, and those who filled a prescription for one of the study drugs that was prescribed by a non-OPEM physician (i.e., by an Endocrinologist), were excluded. Individuals who died before the intervention was delivered were also excluded. Lastly, individuals who were matched to a physician with missing personal identifiers were excluded.

Individuals who received a prescription for an ACE inhibitor, “other” antihypertensive agent (described below), or a cholesterol-lowering agent during the pre-intervention period were identified in ODB. The prescription(s) closest to January 15, 2005 (PEM mailout) were identified and the OPEM physician/physician group that prescribed these drugs was flagged. The patient was then linked to this physician/physician group. If a patient was not prescribed a study drug during the pre-intervention period, this patient was linked to the OPEM physician/physician group that provided the majority of visits for that patient in the pre-intervention year.

### Interventions

*Informed* was a peer-reviewed practice synopsis introduced in 1994 that provided an overview of the latest research findings to promote evidence-based medicine, developed using expertise from both clinical and research staff at ICES [4]. Subscription to *informed* was free and voluntary for all FPs in Ontario, and approximately 15,000 FPs subscribed throughout its 13-year life [18, 19].

**Fig. 1:**
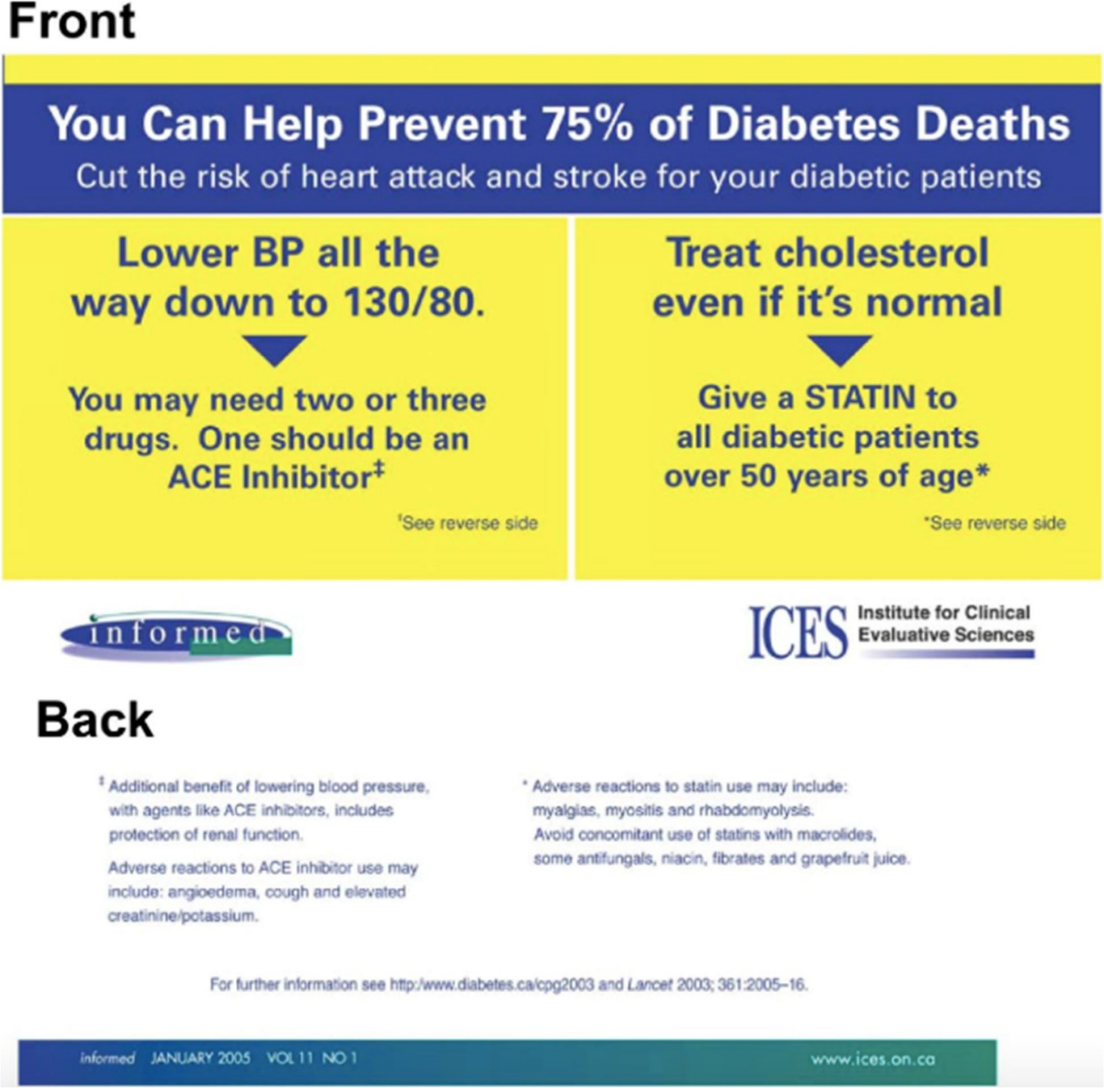
Front and back sides of the outsert PEM

**Fig. 2:**
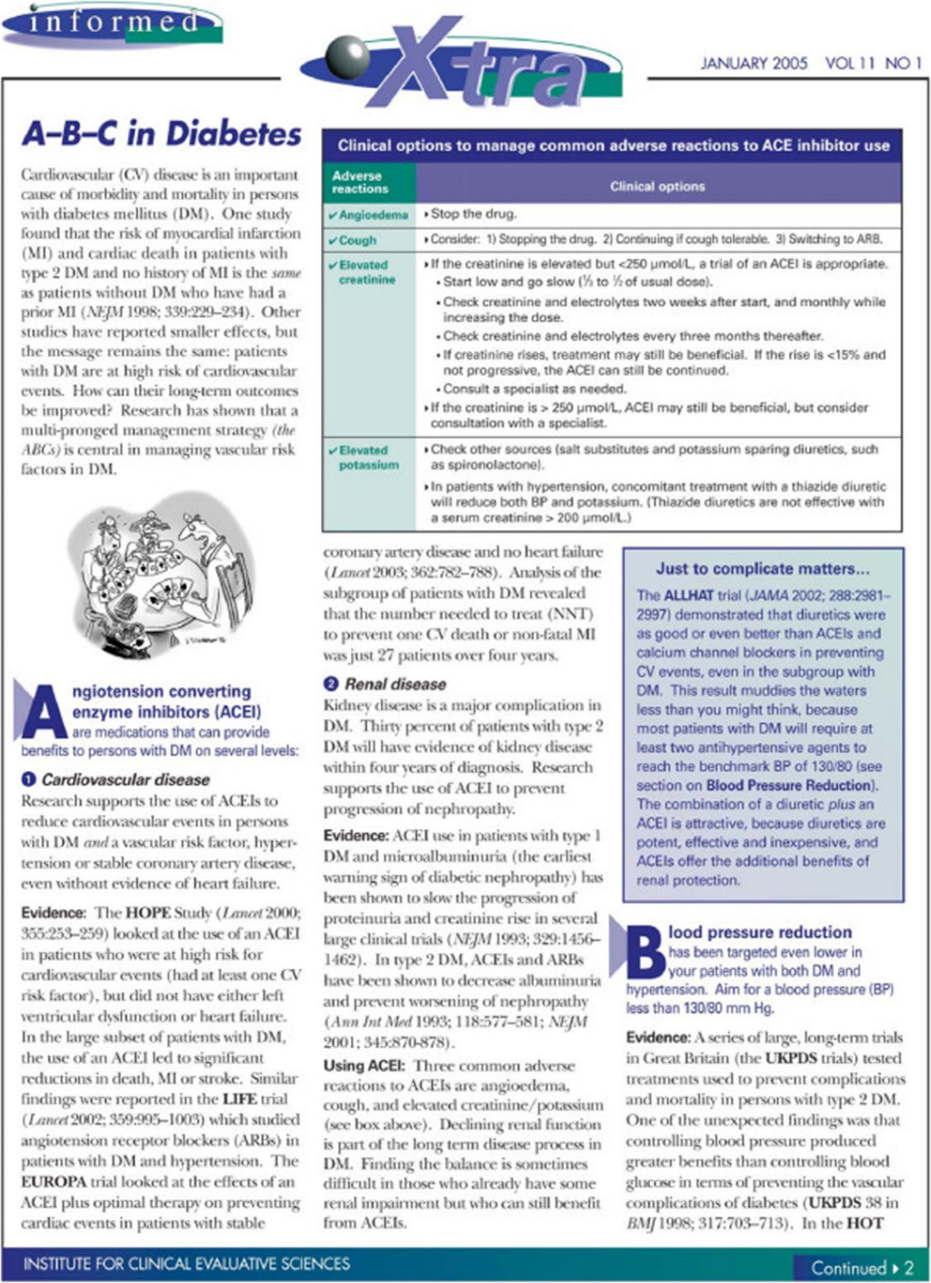
First page of the insert PEM

The OPEM programme utilized the reach of *informed*, and its reputation, to deliver the intervention to the eligible FPs. The PEMs came in two different forms: a short, directive message (outsert), and a long, detailed message (insert) (see Figs. 1 and 2, respectively). The outsert was a postcard-sized paper attached to the front page of *informed* in the bottom left corner [4]. Bright colors and large font sizes were used to attract the eye of the reader. The main recommendations to prescribe more than one antihypertensive, one of which is an ACE inhibitor, and a cholesterol-lowering agent are clearly highlighted on front side of the outsert, while the back side provides a brief explanation for the recommendations, as well as a link to obtain more information [4]. The alternative intervention against which the outsert was compared was a two-page, more traditional narrative review article (insert) that was designed to look like the rest of the articles in that edition of the newsletter, but discussed a topic that was not covered elsewhere in the edition [4]. FPs received one of the two messages, both, or neither; all four groups received the same issue of *informed*. PEMs were only delivered once.

### Outcomes

The goal of the trial was to determine whether PEMs are effective at prompting physicians to intensify their patient’s treatment regimen. A standard definition for the intensification of diabetes treatment does not exist. However, the addition of a new (i.e., not previously prescribed) agent to the patient’s treatment regimen and an increase in dose of an existing medication are often used as indicators [26–29].

#### Primary

The primary outcome is therefore defined as the intensification of a patient’s treatment regimen by adding a new drug, either an angiotensin-converting enzyme (ACE) inhibitor, “other” antihypertensive (e.g., angiotensin II receptor blockers, beta-blockers, calcium channel blockers, diuretics), or cholesterol-lowering agent (e.g., statins, fibrates, antilipemic and atherosclerosis inhibiting agents) and/or increasing the dose of a current ACE inhibitor, “other” antihypertensive, or cholesterol-lowering agent (see Additional file 1 Table 2 for a detailed description of outcome programming).

#### Secondary

Drug switches occur for a multitude of reasons, including patient preference, physician preference, pharmaceutical influence, and patient side effects. Physicians may have switched their patient from one drug to another, perhaps a stronger drug, in response to the intervention. Thus, medication switches were included in the secondary outcome measurement to capture physicians who intensified their patient’s treatment regimen by making a drug switch. The secondary outcome is therefore defined as the addition of an ACE inhibitor, “other” antihypertensive agent, or cholesterol-lowering agent, the increase in dose of a current ACE inhibitor, “other” antihypertensive agent, or cholesterol-lowering agent, or the switch from one drug to another across all drug classes.

### Power

We were interested in detecting improvements in the proportion of individuals experiencing treatment intensification as small as 5% [4]. Previous data revealed that 36% of individuals with diabetes in Ontario were prescribed an ACE inhibitor at baseline [4]. With an assumption that each FP sees ten individuals with diabetes, and an intracluster correlation coefficient of 0.1, 1250 practices per arm provides 97% power to detect a 5% improvement in prescribing [4].

### Statistical analysis

Logistic regression models estimated using generalized estimating equations with robust standard errors were fit to ascertain the effectiveness of PEMs while taking into account the clustering of patients to their physician groups. Analyses followed the recommendations set forth by the CONSORT extension for cRCTs [30]. Further, following standard practice for the analysis of pragmatic RCTs, the primary and secondary analyses were planned as intention-to-treat analyses. Three logistic regression models were fit: two for the primary outcome (unadjusted, and adjusted for physician characteristics), and one for the secondary outcome (unadjusted). We deemed two-tailed *p*-values less than or equal to 0.05 to be statistically significant. All statistical analyses were conducted using SAS version 9.4 (SAS Institute, Cary, NC).

## Results

### Physician and patient selection

**Fig. 3:**
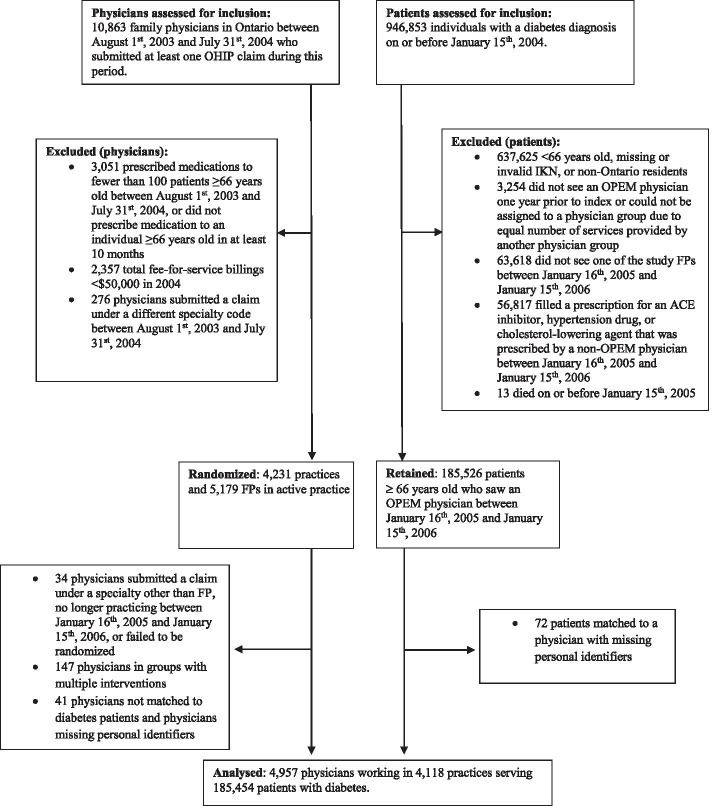
Physician and patient selection

The number of physicians and patients included in the trial is shown in Fig. 3. Over half of FPs were deemed ineligible due to not being in “active” practice or for submitting a claim under another speciality during the lookback period. A further 221 were excluded after randomization for reasons such as submitting a claim under a different specialty, no longer practicing, and working in a practice that received multiple PEM formats during the post-intervention period; thus, 4957 physicians were ultimately included, working in 4118 practices. Approximately 20% of individuals with a diabetes diagnosis on or before January 15, 2004 met the eligibility criteria. Seventy-two patients were excluded after randomization because they were matched to a physician with missing information (ICES constraints); accordingly, 185,454 patients were included in the final analysis.

### Descriptive statistics

1025, 1037, 1031, and 1025 physician practices (clusters) received the insert, outsert, both, and neither, respectively, and were analysed. There were 44,845, 47,602, 45,508, and 47,499 patients in physician practices that received the insert, outsert, both, and neither, respectively. Characteristics of the physicians and the patients at baseline were well balanced (Tables 1 and 2). The number of physicians working in solo and shared practices was 3548 (72%) and 1409 (28%), respectively. The median number of eligible diabetes patients per physician group was 36 (interquartile range (IQR) = 38), while the median number of patients per individual physician was 32 (IQR = 31).

**Table 1:** Baseline characteristics of physicians

Variable	Statistic	***informed*** only	***informed*** + insert	***informed*** + outsert	***informed*** +insert + outsert
	N (%)	1254 (25.3)	1228 (24.8)	1255 (25.3)	1220 (24.6)
Age (years)	Mean ± SD	52.1 (10.0)	51.9 (10.1)	52.2 (9.9)	52.3 (10.3)
Sex	Female (%)	260 (20.7)	257 (20.9)	283 (22.5)	300 (24.6)
Canadian Medical Graduate	Yes (%)	951 (75.8)	951 (77.4)	945 (75.3)	941 (77.1)
Years in practice	Mean ± SD	22.0 (9.2)	21.7 (8.8)	22.0 (9.2)	22.2 (9.1)
Visits per year	Mean ± SD	7142.4 (3391.6)	7096.0 (3217.0)	7229.0 (3335.8)	7075.7 (3239.0)
Billings per year (per CAD $10^5^)	Mean ± SD	2.2 (1.0)	2.2 (1.0)	2.3 (1.1)	2.2 (1.0)
Practice location	Northern Ontario^a^ (%)	77 (6.1)	99 (8.1)	117 (9.3)	105 (8.6)
Rural practice	Yes^b^ (%)	151 (12.0)	150 (12.2)	150 (12.0)	169 (13.9)

*SD* Standard deviation; “N” is used to denote the total number of individuals in each intervention group

Brackets within the table represent either SD or an overall percent (see “Statistic” column)

^a^ Northern Ontario practices defined by a postal code with a forward sortation area beginning with “P”

^b^ Rural practices defined by practices in locations with a population of less than 10,000

**Table 2:** Baseline characteristics of patients

Variable	Statistic	***informed*** only	***informed*** + insert	***informed*** + outsert	***informed*** + insert + outsert
	N (%)	47,499 (25.6)	44,845 (24.2)	47,602 (25.7)	45,508 (24.5)
Age (years)	66–74 (%)	22,895 (48.4)	21,469 (47.9)	22,909 (48.1)	22,096 (48.6)
	75–84 (%)	19,603 (41.3)	18,599 (41.5)	19,790 (41.6)	18,914 (41.6)
	85+ (%)	4911 (10.3)	4777 (10.7)	4903 (10.3)	4498 (9.9)
Sex	Female (%)	24,615 (51.8)	23,364 (52.1)	24,879 (52.3)	23,695 (52.1)
Years since diabetes diagnosis	Mean ± SD	7.8 (4.1)	7.8 (4.1)	7.8 (4.1)	7.8 (4.1)
Recent diabetes diagnosis (≤2 years)	Yes (%)	3411 (7.2)	3126 (7.0)	3488 (7.3)	3178 (7.0)
ACE inhibitor use	Yes (%)	28,836 (60.7)	27,114 (60.5)	28,916 (60.8)	27,568 (60.6)
“Other” antihypertensive use	Yes (%)	38,474 (81.0)	36,224 (80.8)	38,452 (80.8)	36,807 (80.9)
Cholesterol lowering agent use	Yes (%)	30,476 (64.2)	28,381 (63.3)	30,213 (63.5)	28,801 (63.3)

*SD* Standard deviation, *ACE inhibitor* Angiotensin-converting enzyme inhibitor; “N” is used to denote the total number of individuals in each intervention group

Brackets within the table represent either SD or an overall percent (see “Statistic” column)

### Analysis of intervention effects: primary outcome

The percent of patients experiencing treatment intensification was similar across trial arms, with approximately 46% receiving an additional drug to their regimen or an increase in dose of a current drug (Table 3). In the main effects model, the odds ratio (OR) for the outsert effect was 1.01 (95% confidence interval (CI) 0.98 to 1.04), and the OR for the insert effect was 0.99 (95% CI 0.96 to 1.02) (Table 4). A model was also fit to include the interaction between the outsert and the insert. The OR for the interaction was 1.01 (95% CI 0.98 to 1.11), indicating that the effect of the outsert was unchanged by the presence of the insert (*p* = 0.17). Further, neither a statistically significant outsert effect, nor an insert effect, were observed after adjusting for the following physician characteristics: physician age, sex, country of medical training (Canadian vs. other), practice location (Northern vs. Southern, rural vs. non-rural), total general patient visits per year, total billings per year, and years in practice (Table 4).

**Table 3:** Percent of patients who experienced treatment intensification

	***informed*** only (1025 clusters)	***informed*** + insert (1025 clusters)	***informed*** + outsert (1037 clusters)	***informed*** + insert + outsert (1031 clusters)
By addition or dose increase^a^	46.4	45.5	46.0	46.2
By addition, dose increase, or switch^b^	48.7	47.9	48.4	48.7

^a^Primary outcome

^b^Secondary outcome

**Table 4:** Unadjusted and adjusted intervention effects

*Intervention*	OR (95% CI)	*P* value
**Regression model 1a: unadjusted effect of insert and outsert (primary outcome)**		
Outsert	1.01 (0.98, 1.04)	0.74
Insert	0.99 (0.96, 1.02)	0.50
**Regression model 1b: effect of insert and outsert, adjusted for physician characteristics**		
Outsert	1.00 (0.97, 1.03)	0.77
Insert	0.99 (0.96, 1.02)	0.58
*Physician characteristics*		
Physician age (per 10 years)	1.00 (0.97, 1.04)	0.94
Sex (reference is male)	1.08 (1.03, 1.12)	< 0.0001
Canadian medical graduate (reference is no)	0.93 (0.89, 0.97)	< 0.0001
Years in practice (per 10 years)	0.96 (0.91, 0.99)	0.03
Total visits (per 100 visits)	1.00 (1.00, 1.00)	0.23
Total billings (per $10,000)	1.00 (1.00, 1.00)	0.10
Practice location (reference is Southern)	0.97 (0.91, 1.03)	0.32
Rural practice (reference is no)	0.99 (0.94, 1.03)	0.55
**Regression model 2: unadjusted effect of insert and outsert (secondary outcome)**		
Outsert	1.01 (0.98, 1.04)	0.60
Insert	0.99 (0.96, 1.02)	0.64

The intracluster correlation coefficient was 0.023, and the variance inflation factor was 2.01.

### Analysis of intervention effects: secondary outcome

The percent of individuals experiencing a treatment intensification based on additions, dose increases, and switches was comparable across treatment groups and was, on average, slightly higher (approximately 2%) than those based on additions and dose increases alone (Table 3). The ORs for the outsert and insert effects were 1.01 (95% CI 0.98 to 1.04) and 0.99 (95% CI 0.96 to 1.02), respectively (Table 4).

## Discussion

The aim of the OPEM trial was to determine whether PEMs can successfully influence FPs to improve adherence to guideline recommendations for diabetes care through treatment intensification. There were no changes (neither statistically, nor clinically) in intensification of medications (with or without including drug switches) in response to either version of the PEM, with no interaction effect. Thus, while the trial was primarily designed to test the independent effects of both the outsert and the insert, we have little reason to believe that FPs who received the combination of both PEMs would have any difference in outcome. The PEMs are mostly duplicates of each other; FPs, who already face demands on their time, are unlikely to read the insert in full after reading the brief, post-card sized summary.

Over all arms, regardless of the intervention received, we found that female physicians, those who received their medical training outside of Canada, and those who had been in practice for fewer years were more likely to engage in treatment intensification (Table 4). Nevertheless, these three effect sizes were very close to one, suggesting that, while statistically significant, the effect of the aforementioned physician characteristics on treatment intensification was minimal and is unlikely to translate into clinically meaningful effects.

Our findings are consistent with previous research [13, 16, 31, 32], including our own previously published OPEM trials [18, 19]. The failure of PEMs to bring about behavior change among physicians may be an indication that this KT strategy is largely ineffective and could be safely abandoned. PEM-based interventions are centered around the idea that evidence, once shared with an individual, prompts change. The negative findings from this and many other studies suggests that other barriers may exist. For example, physicians often struggle to “unlearn” old behaviors [33, 34]. To change clinical practice, physicians must replace routine, outdated operations with new, evidence-based practices. While adding to their knowledge base can be a simpler undertaking, dismissing current practices presents a greater challenge [33, 34]. The process of change disrupts the status quo equilibrium, causing physicians to be uncertain about practices they considered to be “certain” [34]. Physicians included in the study had been practicing as an FP for 22 years on average (Table 1). As a result, it is possible that they may have adopted standard prescribing practices for individuals with diabetes over the years and were thus hesitant to implement a change to multiple drug classes when presented with evidence in the PEMs. Furthermore, the patients themselves may act as a barrier to PEM effectiveness. A qualitative study by Parsons et al. [35] investigated why printed educational toolkits mailed to Ontario FPs failed to improve cardiovascular disease management among individuals with diabetes. The FPs believed that patient motivation and willingness to change behavior was the primary obstacle preventing them from adhering to the PEM recommendations [35].

### Strengths

Our trial was randomized, and, as consent was not required, also blinded with high internal validity. It was also very large, offering a precise estimate of effect, and was designed with mostly pragmatic features to align with our pragmatic (decision-making) intention, increasing its relevance to real-world decision making in Ontario and similar settings (see Additional file 1 Table 1 and Fig. 1 for PRECIS-2 table and wheel, respectively) [36]. The PEMs were delivered in a way that is likely similar to that of a routine large scale programme: by mail to physicians alongside a journal many of them routinely received, targeting the providers who usually care for patients with diabetes in Ontario. Outcomes were measured using routinely collected electronic health information, avoiding any distortions to usual care that might arise from data collection from physicians or patients. Lastly, while prescribing recommendations were outlined in the PEMs, the choice to prescribe was ultimately left to the physician – a naturalistic approach with no restrictions on behaviour, identical to a PEM program as might be implemented in routine practice.

### Limitations

Our study design cannot distinguish the point at which PEMs failed. Failure may be due to non-delivery, or the messages not being opened or read by the physicians. A 1997 survey revealed that Ontario physicians considered *informed* to be a useful source of clinical information (71% of physicians reporting received *informed*, and of these, 89% reported that the information was useful) [37]. But receptiveness may not be the same as clinical influence, and 8 years later, at the time of our trial, its influence may have declined. Further, while Canadian postal delivery is reliable, physicians may not be opening their own mail, and even if they do, may not be reading *informed*.

Our study is relevant only to print based educational materials delivered by mail. It is possible that physicians would have been more influenced by the PEM had it been delivered by an alternative route (e.g., fax, email). Following the shift towards electronic information as a basis for clinical decision making, electronically-delivered recommendations may have been more widely received by physicians [38, 39]. However, these are seldom reported in the literature. A 2020 systematic review of PEMs identified only two studies comparing printed versus electronically delivered guidelines and found little to no difference in effectiveness between the two delivery strategies [17]. That said, the quality of evidence from these studies was graded as low [17]; thus, additional studies investigating electronically delivered guidelines may be warranted.

While the trial was designed with a pragmatic intention, it focused on higher volume clinicians treating older persons with diabetes. Only 5179 of the 10,863 eligible FPs practicing in Ontario were included, and although the excluded physicians were mostly inactive or saw few patients over 65 years, we cannot claim complete representativeness. The results are applicable to Ontario FPs who billed at least $50,000 in 2004, and who prescribed medications to at least 100 seniors (≥66 years old) in at least 10 months in 2004. It is possible that physicians who treat fewer individuals 66 and above and have less experience with diabetes care might have a different outcome.

In addition, the ODB claims database only contains prescriptions that have been reimbursed, which depends on whether they have been dispensed. The intervention effect might have been diluted by patients who received an intensified prescription but failed to get it dispensed. These individuals are likely to be balanced among the four groups as a consequence of randomization; thus, our estimate of the relative effect is unlikely to biased.

A discussion of the limitations would be incomplete without acknowledging the timeframe in which the study was conducted. The PEMs were mailed in January 2005, and the outcome measurement took place between 2005 and 2006. Estimates at the time of the trial suggested that prescriptions for all of ACE inhibitors, “other” antihypertensives, and cholesterol-lowering agents were at least 30% below guideline recommendations [4]. One may wonder whether the results from the present study are relevant to clinical practice today. In 2015, it was found that the percent of patients aged 65 and above with diabetes in Ontario who filled a prescription for an ACE inhibitor or angiotensin II receptor blocker and a statin was approximately 60 and 72%, respectively [40]. Further, the 2018 Diabetes Clinical Practice Guidelines still declare that diabetes-related cardiovascular complications are responsible for the greatest burden of death among individuals with diabetes and recommend treatment with appropriate medications to manage these risks, including antihypertensives and cholesterol-lowering agents [11]. Thus, the evidence-to-practice gap continues to persist. It is, however, worth noting that, while the recommended target drug classes remain the same, some of the medications that were included as part of treatment intensification in the trial, for example atherosclerosis inhibiting agents, are not recommended today. Therefore, one must interpret the results within the timeframe of the study.

## Conclusions

Overall, in this large, randomized trial, we found that PEMs alone, whether long and narrative or brief and action focused are not effective at intensifying prescriptions for individuals with diabetes aged 66 and above in Ontario primary care. The implications for local decision makers are clear: despite their low costs and wide reach, PEMs, in the formats we tested, are not effective at bridging diabetes-related evidence-to-practice gaps in primary care in Ontario. Funding agencies should be cautious in supporting additional evaluations of these PEMs without first developing a deep and theoretically coherent understanding of the facilitators and barriers to their success. Researchers may consider conducting qualitative studies to gain perspective from the physicians on why this particular KT strategy has been repeatedly found to be ineffective, and to determine whether modifications to the design of the PEMs and/or the method of delivery may result in greater uptake, or, whether this KT strategy should be safely abandoned. In this modern era of electronic health records, one might look towards electronic interventions such as point of care reminders, on screen prompts, and electronic audit and feedback [41, 42].

## Supplementary Information

**Additional file 1.** PRECIS-2 table, wheel, and outcome programming.

## Data Availability

The dataset from this study is held securely in coded form at ICES. While data sharing agreements prohibit ICES from making the dataset publicly available, access may be granted to those who meet pre-specified criteria for confidential access, available at www.ices.on.ca/DAS. The full dataset creation plan and underlying analytic code are available from the authors upon request, understanding that the computer programs may rely upon coding templates or macros that are unique to ICES and are therefore either inaccessible or may require modification.

## Abbreviations

ACE inhibitor: Angiotensin-converting enzyme inhibitor
CPDB: Corporate Provider Database
cRCT: Cluster randomized controlled trial
DIN: Drug identification number
FP: Family physician
IPDB: ICES Physician Database
IQR: Interquartile range
KT: Knowledge translation
ODB: Ontario Drug Benefit Claims
ODD: Ontario Diabetes Dataset
OHIP: Ontario Health Insurance Plan Claims Database
OPEM: Ontario Printed Educational Message programme
OR: Odds ratio
PEM: Printed educational material
RCT: Randomized controlled trial
RPDB: Registered Persons Database
SD: Standard deviation
