# Supplementary material for: Printed educational materials directed at Ontario family physicians do not improve adherence to guideline recommendations for diabetes management: a pragmatic, factorial, cluster randomized controlled trial [ISRCTN72772651]
Source: BMC Fam Pract. 2021 Dec 11;22:243. doi: 10.1186/s12875-021-01592-9 (PMC8666060; doi:10.1186/s12875-021-01592-9)
Supplement: Supplementary file 1 — Additional file 1. PRECIS-2 table, wheel, and outcome programming. [file 12875_2021_1592_MOESM1_ESM.docx]

**Additional file 1**

Table 1: PRECIS-2 design features of the OPEM diabetes trial.

| PRECIS-2 domain | Score | Explanation |
| --- | --- | --- |
| Eligibility | 4 | - Physicians: Almost all Ontario FPs in “active” practice during the trial period - Patients: Almost all individuals 66 and above with type 1 and type 2 diabetes in Ontario |
| Recruitment | 5 | - Physicians and patients identified from administrative databases held at ICES ­­– no consent required, zero impact on behavior |
| Setting | 5 | - FP practices across Ontario, Canada – no exclusions based on geography, staffing levels, patient population, etc. |
| Organization | 5 | - No additional staff or training required to deliver the intervention – unobtrusive and feasible to do unchanged in usual Ontario setting, provided the ministry or other organization develops and mails the intervention |
| Flexibility: delivery | 5 | - Guideline recommendations are provided to physicians in the PEMs, but the choice to prescribe is ultimately up to the physician – a naturalistic approach with no restrictions on behavior so would be identical if implemented as policy |
| Flexibility: adherence | 5 | - No measures in place to monitor whether physicians receive, open, and read the PEM |
| Follow-up | 5 | - One-year post intervention mailout by means of administrative databases – no contact at all with individual physicians or patients |
| Primary outcome | 4 | - Behavior change among physicians (manifested through intensification of prescriptions for ACE inhibitors, “other” antihypertensives, and cholesterol-lowering agents) – more important to physicians than to patients |
| Primary analysis | 5 | - Intention-to-treat analysis; no physicians or patients lost to follow-up |


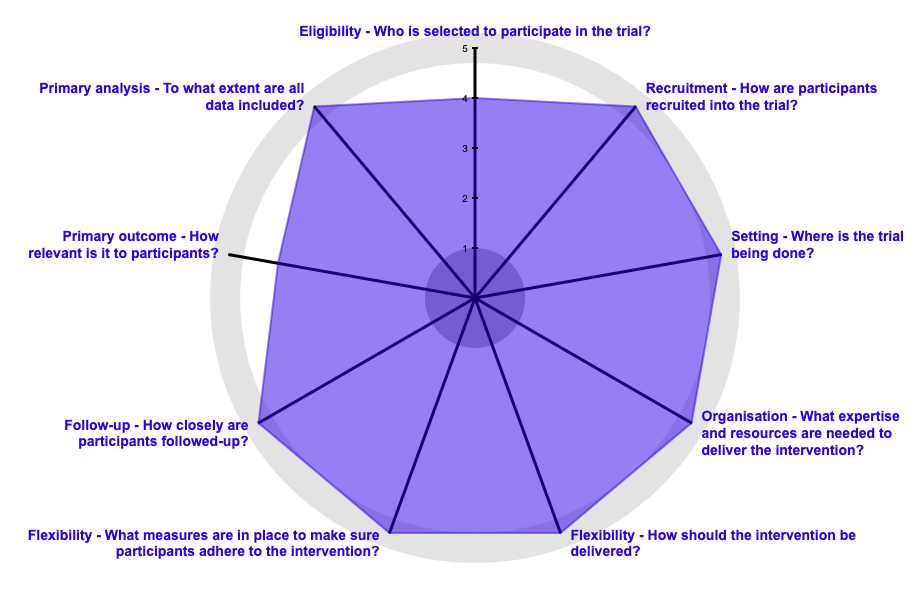
Figure 1: PRECIS-2 wheel for the OPEM diabetes trial.

Table 2: Outcome programming

| Pre-intervention prescription list | 1. Pull all drugs from classes of interest. 2. Ensure patient is on each drug as of index date (January 15, 2005) and that the GP group is the same as the GP group to which they were linked. 3. If there are multiple drugs with the same name (‘drug name’ in drug list), choose the DIN within that drug name that is closest to index. 4. Calculate dose of drugs (calculation below). |
| --- | --- |
| Post-intervention prescription list | 1. Pull all drugs from classes of interest. 2. Ensure drugs were prescribed by an OPEM physician. 3. Calculate dose of drugs. |
| **PRIMARY OUTCOME** | |
| Definition | The intensification of medications for controlling cardiovascular complications associated with diabetes. For a therapeutic regimen to be considered to be intensified, the FP must complete one of the following in the 1-year following the receipt of a PEM:   1. Add an ACE inhibitor, “other” antihypertensive agent, or cholesterol-lowering agent; or 2. Increase the dose of a current ACE inhibitor, “other” antihypertensive agent, or cholesterol-lowering agent. |
| Programming | **Drug addition**:   - Number of drugs post-intervention>number of drugs pre-intervention (if npost>npre, then addition=1)   **Dose increase** (where dose=(quantity*strength)/day supply):   - Increase in dose of the same drug (‘drug name’ in drug list) (if dose of ‘drug name’ post-intervention > dose pre-intervention, then dose increase=1) |
| **SECONDARY OUTCOME** | |
| Definition | The intensification of medications for controlling cardiovascular complications associated with diabetes. For a therapeutic regimen to be considered to be intensified, the FP must complete one of the following in the 1-year following the receipt of a PEM:   1. Add an ACE inhibitor, antihypertensive agent, or cholesterol-lowering agent; or 2. Increase the dose of a current ACE inhibitor, antihypertensive agent, or cholesterol-lowering agent; or 3. Switch from one drug to another (irrespective of drug class; allows a patient to switch from an antihypertensive to an ACE inhibitor, etc.) |
| Programming | See primary outcome for programming of additions and dose increases  **Switch**  If number of drugs pre- and post-intervention is the same, and the patient has a ‘drug name’ post-intervention that wasn’t in their pre-intervention drug list, then switch=1 |
